# Development and utility of an in vitro, fluorescence-based assay for the discovery of novel compounds against dengue 2 viral protease

**DOI:** 10.1186/s41182-016-0025-6

**Published:** 2016-08-10

**Authors:** Gianne Eduard L. Ulanday, Kenta Okamoto, Kouichi Morita

**Affiliations:** 1 Department of Virology, Institute of Tropical Medicine, Nagasaki University, 1-12-4 Sakamoto, Nagasaki, 852-8523 Japan; 2 Graduate School of Biomedical Sciences, Nagasaki University, Nagasaki, Japan; 3 Leading Graduate School Program, Nagasaki University, Nagasaki, Japan

**Keywords:** Dengue virus, Antiviral screening, Dengue protease, NS2B-NS3pro

## Abstract

**Background:**

Dengue disease is one of the most significant vector-borne illnesses in the world. The emergence and re-emergence of dengue infections in many parts of the world affect millions annually and continue to burden public health systems especially in low-income populations. Advances in dengue vaccine development showed promising results; however, protection seems to be suboptimal. There is no licensed chemotherapeutic agent against dengue to date. An ideal scenario of combinatorial vaccination of high-risk individuals and chemotherapy of the diseased during outbreaks may compensate for the meager protection offered by the vaccine. The dengue virus protease is important to viral replication and, as such, has been identified as a potential target for antivirals. It is, therefore, our objective to establish and optimize an appropriate screening method for use during the early stages of drug development for dengue.

**Methods:**

In this study, we developed and optimized a biochemical assay system for use in screening compound libraries against dengue virus protease. We tested the selected protease inhibitors with a cell-based assay to determine inhibition of viral replication.

**Results:**

We have presented direct plots of substrate kinetics data showing an apparent inhibition of the protease at excessive substrate concentrations. The most common sources of interference that may have affected the said observation were elucidated. Finally, a screen was done on an existing compound library using the developed method. The compounds selected in this study showed inhibitory activity against both the recombinant dengue protease and cell-based infectivity assays.

**Conclusions:**

Our study shows the practicality of a customized biochemical assay to find possible inhibitors of dengue viral protease during the initial stages of drug discovery.

**Electronic supplementary material:**

The online version of this article (doi:10.1186/s41182-016-0025-6) contains supplementary material, which is available to authorized users.

## Background

Dengue disease is a public health problem that continues to affect approximately 100 countries globally with an estimated 390 million cases per year of which majority are children. The burden of disease lies mostly in the Asia-Pacific yet the number of affected countries continues to rise as it emerges into new areas putting more people at risk [1]. Trans-ovarial transmission of dengue virus (DENV) within mosquito populations can facilitate transmission between or within epidemics, thus complicating disease control strategies [2, 3]. Human migration and vector invasiveness contributed to the emergence and re-emergence of dengue in Europe and Asia thus highlighting the need for a more proactive approach in dealing with this neglected disease [4, 5].

The causative agent, dengue virus, is a single-stranded RNA virus belonging to the *Flavivirus* genus of the family *Flaviviridae*. It has four serotypes wherein each DENV serotype is phylogenetically distinct, suggesting that each could be considered as a separate virus. Transmission is accomplished through the bite of mosquitoes, prominently, *Aedes aegypti* and *Aedes albopictus*. In humans, intrinsic incubation period ranges 3–10 days after the bite of infected mosquitoes [6, 7]. Although dengue infection is generally asymptomatic, it may result in a wide spectrum of clinical disease, ranging from a mild flu-like syndrome (dengue fever) to the most severe forms of the disease, which are characterized by coagulopathy, increased vascular fragility, and permeability (dengue hemorrhagic fever). The latter may progress to hypovolemic shock (dengue shock syndrome). Poor clinical outcomes have been linked to secondary infections [8]. However, not all heterologous secondary infections cause subsequent severe forms of the disease. Host and viral factors, age, and timing of infection are some of the aspects to be considered [9].

The lack of chemotherapeutic agents and heavy reliance on vector control complicate the management and prevention of dengue. Although recent advances in vaccine development have proven that a tetravalent preparation is possible, field trials showed only around 50 % vaccine efficacy and meager protection against specific serotypes [10, 11]. One study estimates vaccine protection in a population from 24 to 54 % according to their models [12]. This observation led to more questions regarding the mechanisms involving immunity against dengue and proxy determinants of protection. The success of marketed drugs targeting viral enzymes such as in hepatitis C virus (HCV) and human immunodeficiency virus (HIV) has directed the interest of researchers towards flaviviral proteases. Recent literature suggests that the viral proteins of DENV will probably have a lower tendency to experience resistance-causing mutations than in the case of HCV and therefore is suitable as potential drug targets [13].

The 11 kilobase DENV genome encodes a polyprotein containing the structural and nonstructural (NS) proteins. The polyprotein is processed by several proteases, including that of the host. Similar with other flaviviruses, the dengue virus protease is considered as a trypsin-like serine protease located within NS3 [13]. The NS3 has two enzymatic domains, a serine protease (NS3pro) and helicase component located at the amino and carboxyl terminals, respectively. Interestingly, DENV protease has a marked cleavage site preference for dibasic residues which is distinct from native trypsin [14]. The protease domain requires the 40-amino acid central hydrophilic region of NS2B (NS2B(H)) as a cofactor which forms as part of the substrate recognition region [13]. The NS2B(H)-NS3 protease complex (NS2B(H)-NS3pro) is vital for the post-translational processing of the polyprotein precursor and is essential for viral replication and maturation of infectious virions [14]. Considering these points, the dengue protease component located within NS3 is a very promising target for antiviral development. Hence, the present study aims to establish and optimize an assay for use during the early stages of drug development for dengue.

In this study, we have constructed and expressed a recombinant DENV2 viral protease for use in screening of potential lead compounds. Our optimized biochemical assay provided a straightforward and practical alternative to currently available assay methodologies. Also, we have shown and compared two enzyme kinetic models to guide future researchers in analyzing their data. The established assay identified potential lead compounds which were confirmed by cell-based assays.

## Methods

### Cloning of pET28a/DENV NS2B(H)-G4SG4-NS3pro of DENV2

The DENV NS2B(H)-NS3pro gene sequence containing hydrophilic residues 49-96 of NS2B and N-terminal 180 amino acids of NS3 were amplified from DENV-2 16681 infectious clone (GenBank Accession: U87411) (given by Richard Kinney, Center for Disease Control and Prevention, Division of Vector-borne Diseases, Fort Collins, CO, USA) analogous as described previously [15, 16]. Briefly, the primer pairs Nhe1-NS2B-F and (G4SG4)-NS2B-R, (G4SG4)-NS3-F, and Xho1-NS3-R were used during polymerase chain reaction (PCR) to produce the fragments NS2B(H)-Linker and Linker-NS3pro, respectively. Subsequently, overlapping PCR was performed through the combination of both templates with primer pairs Nhe1-NS2B-F and Xho1-NS3-R to produce the final full length NS2B(H)-G4SG4-NS3pro incorporating an overlapping region of 27 nucleotides and restriction endonuclease recognition sites at both ends. The PCR product was then digested with *NheI* and *XhoI* before cloning into pET28a vector (Novagen) to yield the N-terminal polyhistidine-tagged fusion protein. All PCR reactions were done using Phusion® High-Fidelity DNA Polymerase (New England Biolabs). Construct sequence was confirmed by automated Sanger sequencing using BigDye® Terminator v3.1 Cycle Sequencing Kit (Applied Biosystems).

### Expression and purification of DENV2 NS2B(H)-G4SG4-NS3pro (rNS2B3pro)

The pET28a construct containing the DENV NS2B(H)-G4SG4-NS3pro was transformed into BL21-CodonPlus (DE3)-RIPL strain (Agilent) which provides correction for possible codon usage bias. Cells were then grown in 250-mL Luria-Bertani broth containing 50 μg/mL kanamycin until OD_600_ reached 0.6–0.8. Protein expression was induced by adding isopropyl β-d-1-thiogalactopyranoside (Wako, Japan) at 0.5 mM final concentration for 3 h at 37 °C. Cells were then harvested by centrifugation at 7000 rpm for 10 min at 4 °C and the cell pellet was resuspended in 30-mL cold lysis buffer (phosphate-buffered saline) prior to mechanical lysis by sonication on ice. The cell suspension was then centrifuged at 15,000 rpm for 30 min to separate the supernatant from the insoluble fraction. The collected soluble fraction was then filtered through a 0.45-μm polyvinylidene fluoride (PVDF) syringe filter unit (Merck-Millipore).

The filtrate containing the histidine-tagged fusion protein was subsequently purified using gravity flow immobilized metal affinity chromatography (IMAC). Briefly, Cobalt TALON® (Takara-Clontech, Japan) was loaded into an open column to approximately 1-mL bed volume. The column was subsequently washed with 20 volumes of distilled water prior to column equilibration with another 20 volumes of lysis buffer. The previously filtered sample was loaded then washed with 40-mL native wash buffer (20 mM imidazole in lysis buffer) to remove other contaminating proteins. Elution was started upon addition of 5 mL each of 100, 200, 400, and 800 mM imidazole (Wako, Japan) suspended in lysis buffer. The elution fractions were then combined in pairs, 100 and 200 mM, 400 and 800 mM before concentrating by centrifugation using Amicon Ultra-15 (Merck-Millipore). The fraction was further purified using Superdex® 75 (GE Life Sciences, Japan) gel filtration column with running buffer containing 50 mM Tris-HCl (pH 8–9) and 100–300 mM NaCl with a flow rate of 0.4 mL/min. Detection was done using 280 and 220 nm. The different fractions were visualized by Coomassie-stained sodium dodecyl sulfate polyacrylamide gel electrophoresis (SDS-PAGE) gels and anti-histidine Western blots to confirm the presence of the target protein. Protein concentration was determined using Direct Detect® infrared spectrometer (EMD Millipore, USA).

### Confirmation of target protein using SDS-PAGE and immunoblotting (Western blot)

The different elution fractions were collected for the detection of the target protein. Approximately 10 μL of each fraction was loaded for SDS-PAGE analysis and immunoblotting. In Western blotting, PVDF membrane containing the transferred protein was blocked by BlockAce solution (Dainippon Sumitomo Pharma, Osaka, Japan) then treated with mouse anti-histidine monoclonal antibody as primary antibody and sequentially stained by horseradish peroxidase (HRP)-conjugated anti-mouse antibody as secondary antibody. The HRP reaction was developed and measured via chemiluminescence using ImageQuant™ LAS 4000 (GE Life Sciences, Japan) biomolecular imager.

### Validation of protease activity and optimization of assay components

A standard amount of purified protease was added into wells of a black, flat-bottom 96-well plate containing assay buffer (200 mM Tris-HCl, 20 % glycerol, pH 9.5) then mixed. Reaction was started upon the addition of the substrate and was monitored via ARVO MX 1420 Multilabel Counter microplate reader (Perkin-Elmer, Japan) after 30 min. Assays were done at room temperature with a final volume of 100 μL.

A substrate assay was done by comparing five different coumarin-based fluorogenic substrates used in other flavivirus protease studies [17–20]. Briefly, a single concentration (1000 μM) of the selected substrates was added similarly as described above. Reactions were started with the addition of purified recombinant protease (rNS2B3pro) with a final concentration of 100 μg/mL.

It has been mentioned in other studies that glycerol affects the stability of some proteins in suspension; therefore, assay buffer containing various concentrations of glycerol was studied to determine the optimal level to be used in the system [21]. The fluorogenic peptide substrate Bz-nKRR-MCA (Peptides International) was used to monitor enzyme activity.

An inhibitor control is needed to validate screening; therefore, different concentrations of a protease inhibitor, aprotinin, were added into wells containing 100 μg/mL rNS2B3pro and mixed prior to addition of the substrate, Bz-nKRR-MCA. Fluorescence emission was monitored every 5 min for 1 h to determine its stability under assay condition.

Substrate kinetics experiments were done using various concentrations of Bz-nKRR-MCA (10–1000 μM) which were mixed in wells containing an assay buffer. Reaction was initiated upon the addition of purified rNS2B3pro to a final concentration of 1 μg/mL. Substrate hydrolysis was monitored for 30 min using ARVO MX 1420 Multilabel Counter microplate reader (Perkin-Elmer, Japan) at room temperature. To estimate substrate kinetic values, the rates of reaction were plotted as a function of substrate concentration and fitted into an appropriate model.

### Cell-based assays

#### Cell viability assay

Cell viability was determined using CellTiter 96® Non-Radioactive Cell Proliferation Assay (MTT) (Cat #G4000, Promega) according to the manufacturer’s recommendations. Briefly, compounds were resuspended into appropriate concentrations using culture media (Minimum Essential Medium with 2 % fetal calf serum) prior to addition into wells containing Vero cells in confluence. Formazan formation was detected according to protocol after 72 h of incubation at 37 °C with 5 % CO_2_. Two duplicate sample measurements were compared with the vehicle control (1 % dimethyl sulfoxide (DMSO) in culture media) to determine the compound maximum nontoxic dose (MNTD).

### Cell-based infectivity assays

Focus formation assays were done to determine the effect of the compound on viral infectivity. Cells were infected in close analogy to the procedure mentioned previously except that Vero cells were used instead [22]. Serial dilutions of the compound in culture medium were mixed with dengue virus 2 isolated from patient serum, 00-St-022 (GenBank Accession: KF744401.1) then added into wells containing Vero cells. The plates were then incubated at 37 °C with 5 % CO_2_ for 72 h prior to detection of intracellular DENV antigens.

### Antigen detection enzyme-linked immunosorbent assay (ELISA)

ELISA was performed to quantitatively determine the level of viral antigen secreted in culture fluid post-treatment as previously described [22, 23]. Briefly, 96-well microplates were coated with anti-flavivirus IgG from purified pooled serum and then blocked with BlockAce (Dainippon Sumitomo Pharma, Osaka, Japan) in PBS. The 5-day post-infection culture fluids were added, the plates washed, and HRP-conjugated mAb 12D11/7E8 was mixed into wells. The HRP reaction was detected by adding o-phenylenediamine dihydrochloride (OPD) substrate in the presence of 0.02 % hydrogen peroxide, for 30 min at room temperature, and stopped with 1 N sulfuric acid prior to OD measurement at 492 nm.

### Statistical analysis

Analyses were done using GraphPad Prism version 6.07 for Windows, GraphPad Software, La Jolla, California, USA, www.graphpad.com. Estimation of parameters including the 50 % inhibitory concentration (IC_50_) were computed using the above-mentioned software with terms comparable to the recommendations of the International Union of Pharmacology Committee on Receptor Nomenclature and Drug Classification [24, 25]. Statistical treatment of data is specified under each analyses with significance assigned at *p* < 0.05. All experiments were done in duplicates at least twice unless stated otherwise.

## Results

### Cloning, expression, and purification of recombinant DENV2 NS2B3pro

**Fig. 1:**
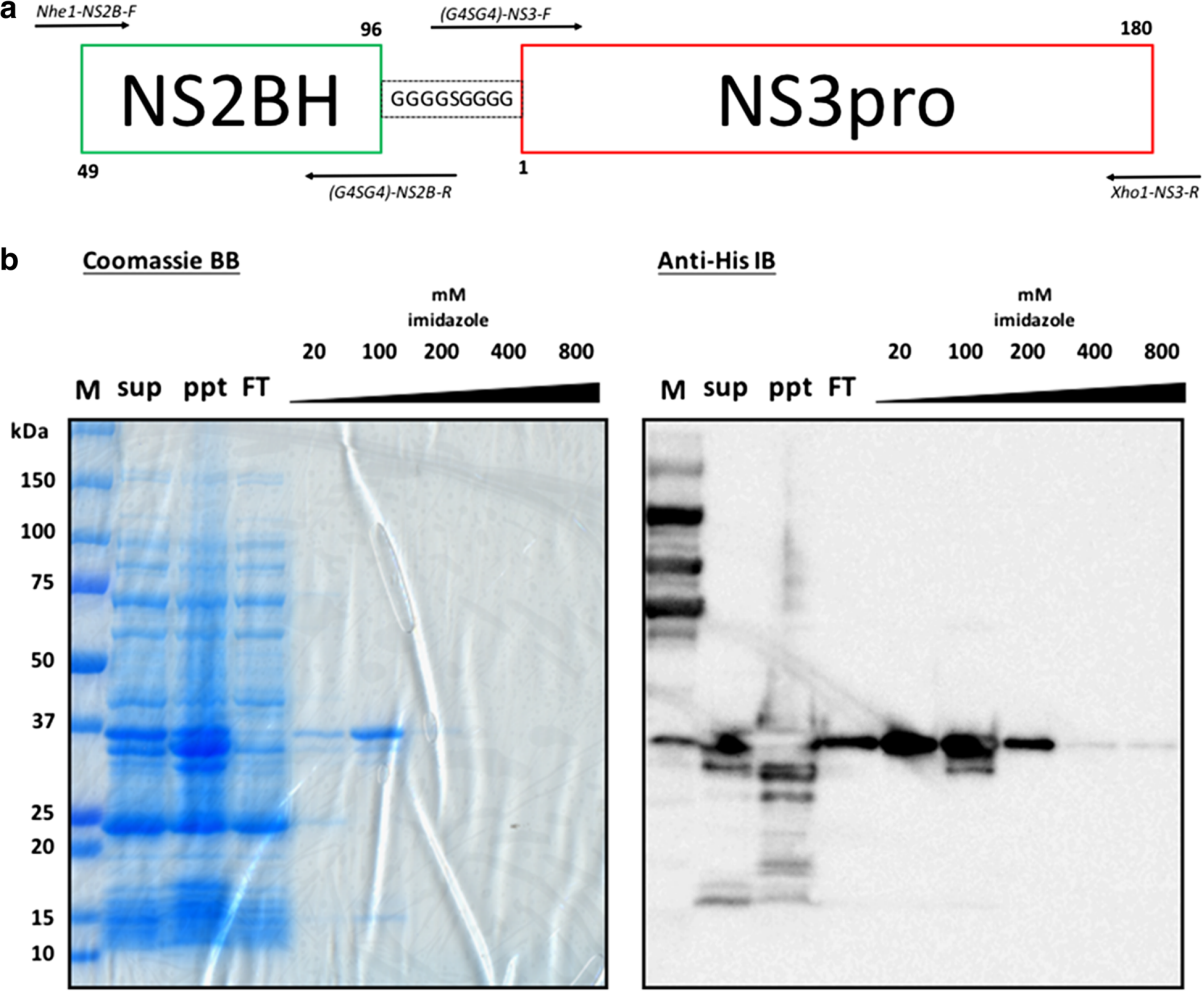
Cloning, expression, and purification of recombinant DENV2 NS2B3pro. **a** Graphical representation of the recombinant protease construct. The figure shows the amino acid positions of the cofactor (NS2BH) and protease complex (NS3pro) linked by a 9-amino acid glycine linker. Binding locations of primers used in the study were indicated. **b** Detection of recombinant protease by Coomassie staining (*left*) and chemiluminescent immunoblotting (*right*). *M* molecular weight marker, *sup* crude supernatant, *ppt* cell pellet, *FT* flow through. Increasing amounts of imidazole eluted the target protein (28 kD) which migrated anomalously at around 37 kD. Both showed elution of the target protein at 100–200 mM imidazole

The DENV2 NS2B(H)-G4SG4-NS3pro plasmid construct included the hydrophilic residues 49-96 of NS2B and N-terminal 180 amino acids of NS3 joined by a glycine linker (Fig. 1a) [26]. The construct sequence was verified by automated sequencing (Sanger method) prior to transformation into expression cells. The 28-kDa recombinant protease (rNS2B3pro) was purified using cobalt immobilized metal affinity chromatography. Different elution fractions were collected and visualized by Coomassie Brilliant Blue staining and anti-histidine immunoblotting. Anomalous migration patterns on SDS-PAGE were observed as in the previous studies (Fig. 1b) [18, 27, 28]. The 100 mM imidazole eluted fraction was further concentrated into approximately 1 mL prior to size exclusion chromatography. Further purification was attempted using Superdex® 75 (GE Life Sciences, Japan) gel filtration column (Additional file 1: Figure S1). Since the HPLC result was comparable to the purity produced by the crude open column IMAC, the latter method was then utilized for the remainder of the study.

### Development of the assay system

Assay conditions mentioned in literature were optimized with emphasis on substrate and control inhibitor selection and additive concentration. Based on the peptide sequence suggested by Li et al. as control, Additional file 1: Figure S2A shows the comparison of different flaviviral fluorogenic substrates at a fixed enzyme concentration as used in other protease studies [26, 27, 29, 30]. The peptide substrate with a sequence norleucine-lysine-arginine-arginine (nKRR) was confirmed to have a higher substrate cleavage activity than that produced by substrates used in other dengue, West Nile, and Japanese encephalitis virus protease assays. The difference in activity produced between the protective groups, benzoyl (Bz) and acetyl (Ac), also seems to be insignificant.

Different concentrations of glycerol were assayed wherein 20 % was considered to be standard in other studies [29, 31, 32]. Truly, the difference in activity among the 0–60 % concentrations can be observed (Additional file 1: Figure S2B) upon comparison against 20 % glycerol.

Assumption of a blank (no substrate) as 100 % inhibition in a compound screen is suboptimal; therefore, a control inhibitor is necessary for screening. Literature search led to the selection of aprotinin for further experiments [29]. Different concentrations of aprotinin were mixed with the recombinant protease prior to fluorescence monitoring every 5 min for 1 h. Additional file 1: Figure S3A shows that at 30 min, a minimum of 40 μM aprotinin was able to inhibit protease activity comparable to that of buffer only controls. The same concentration was able to maintain inhibition up to 1 h (Additional file 1: Figure S3B).

**Fig. 2:**
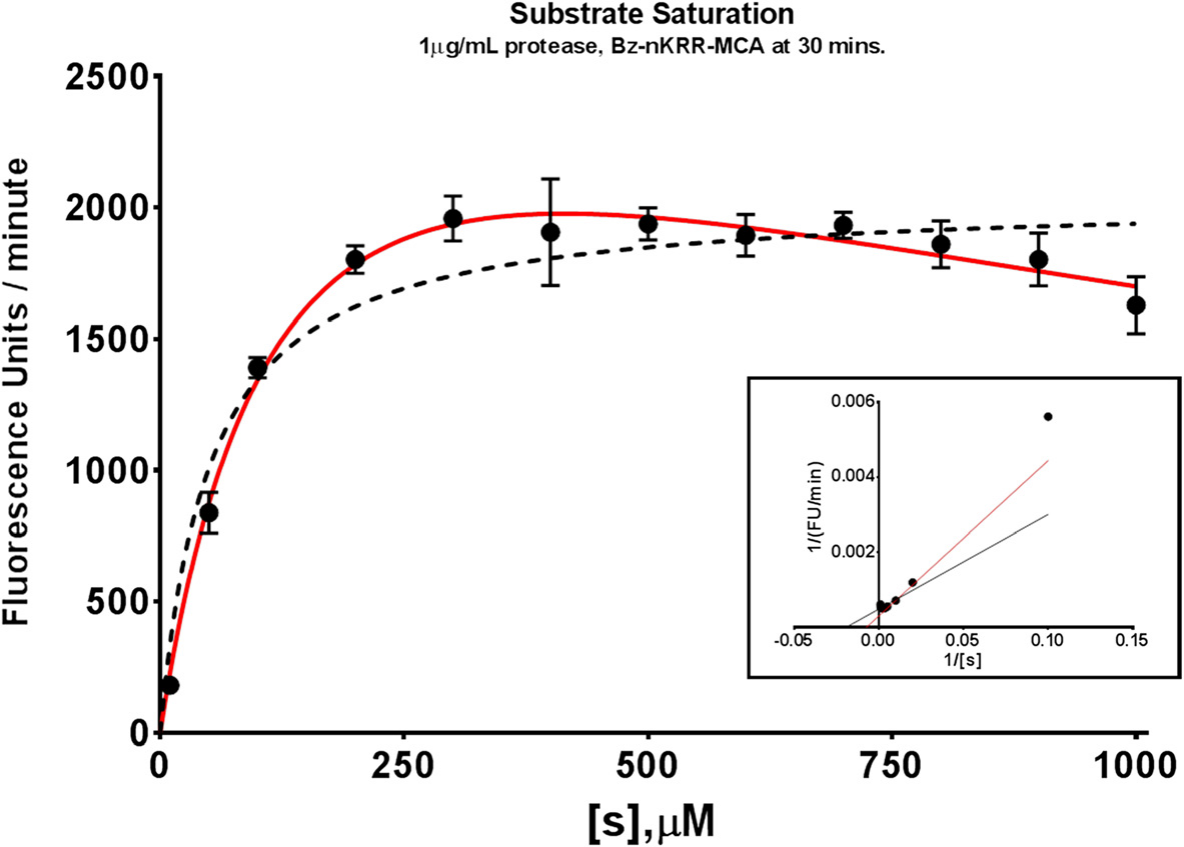
Substrate saturation assay. Fluorogenic substrate was serially diluted prior to addition of 1 μg/mL purified recombinant DENV2 NS2B3pro. Baseline values at time 0 were subtracted. Two nonlinear regression models of enzyme kinetics were plotted; Michaelis-Menten model (*black dashed line*) and substrate inhibition model (*red line*). *Inset* shows Lineweaver-Burk plot of the same. *Error bars* indicate standard deviation (SD)

**Fig. 3:**
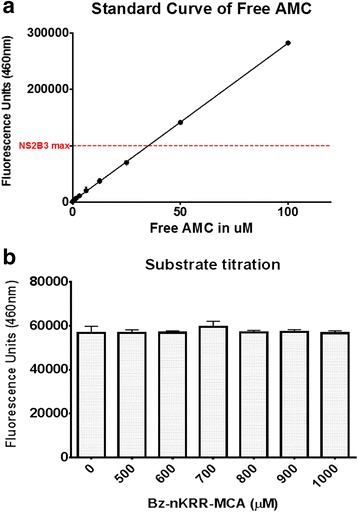
Free fluorophore assay. **a** Serially diluted-free fluorophore (AMC) was evaluated in similar conditions as with the protease assay to determine photodetector saturation. The maximal fluorescence value (approx. 150,000 units) elicited during cleavage conditions was indicated with a *dashed line*. **b** Substrate titration assay. Different concentrations of fluorogenic peptide Bz-nKRR-MCA were mixed with 30 μM of free AMC and read after 30 min of incubation to detect fluorescence quenching. Statistical analysis involved two-way ANOVA with Bonferroni correction. *Error bars* indicate SD

The Michaelis-Menten model requires certain assumptions to be fulfilled for the accurate estimation of kinetic parameters. Firstly, the reaction should be observed at initial velocity conditions, wherein the amount of product formed is negligible to be considered for reversion into the substrate. Figure 2 shows the kinetics of the recombinant protease produced with increasing concentration of the fluorogenic substrate Bz-nKRR-MCA. The protease seems to follow the ubiquitous Michaelis-Menten model kinetics up to around 500 μM of the substrate. However, further increase in the concentration of the substrate led to a model similar to that of substrate inhibition. To further validate the observation, elimination of other extraneous factors such as photodetector saturation, fluorescence quenching, or inner filter effect was necessary. Initially, a fluorophore standard assay was done wherein free 7-amino-4-methylcoumarin (AMC) (Sigma-Aldrich) was serially diluted, processed, and read as mentioned above with the exception of enzyme addition. Photodetector saturation was observed on concentrations at least ten times the maximum fluorescence emitted by substrate cleavage experiments (data not shown). Consequently, the free AMC concentration producing half the maximal values on the standard AMC assay was chosen for the validation assay (Fig. 3a). To determine whether significant absorption of fluorescence emitted by the liberated fluorophore occurred with increasing concentrations of the substrate, simulation of fluorophore release upon hydrolysis was done using free AMC (~30 μM) added into wells containing the uncleaved peptide substrate. Figure 3b shows that there is no significant difference in the levels of fluorescence detected among the groups suggesting that fluorophore quenching by the peptide substrate is highly unlikely. Michaelis constant estimation based on the different models used is presented under Table 1. Results suggest that the model accounting for substrate inhibition fits the data points regardless of maximal substrate concentration.

**Table 1:** Michaelis constant (*K*_*m*_) approximation

	Michaelis-Menten model		Substrate inhibition model	
Max substrate conc.	1000 μM	500 μM	1000 μM	500 μM
*K*_*m*_ in μM (95 % CI)	51.23 (36.80 to 65.66)	76.24 (58.24 to 94.24)	135.0 (99.52 to 170.4)	171.7 (88.73 to 254.6)
Adjusted R square	0.8965	0.9638	0.9687	0.9796

Estimates of *K*_*m*_ derived from varied nonlinear regression models and substrate maximal concentrations at initial velocity conditions were presented

### Selection of compounds

**Fig. 4:**
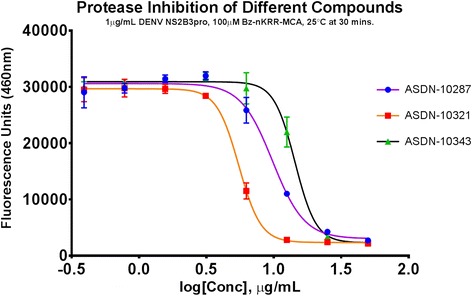
Protease inhibition profile of selected compounds. Serial dilutions of compounds were mixed with 1 μg/mL purified recombinant DENV2 NS2B3pro prior to addition of 100 μM Bz-nKRR-MCA fluorogenic substrate and read after 30 min at 25 °C. *Error bars* indicate SD

The optimized protease screening system based on results mentioned was employed as a duplicate, single concentration (50 μg/mL compound, 1 μg/mL purified protease) assay. Inhibition of protease activity was monitored with fluorogenic peptide substrate, 100 μM Bz-nKRR-MCA, at 30 min using ARVO MX 1420 Multilabel Counter microplate reader (Perkin-Elmer, Japan). Compounds inhibiting at least 85 % of the enzyme control reaction were selected for further investigation. Approximately 500 compounds were screened and three were chosen for further study. Protease IC_50_ values of the three selected compounds ranged from 5 to 14 μg/mL based on the conditions set above (Table 2). Dose response curves plotting concentrations from 50 μg/mL is shown in Fig. 4.

**Table 2:** IC_50_ values of selected compounds. Best-fit values and their respective 95 % confidence intervals are presented

	IC_50_ (μg/mL)	95 % CI (μg/mL)
ASDN-10287	9.786	9.015 to 10.62
ASDN-10321	5.498	5.251 to 5.756
ASDN-10343	14.37	13.26 to 15.57

Determination of was IC_50_ done using nonlinear regression fitting

### Cell viability assay

**Fig. 5:**
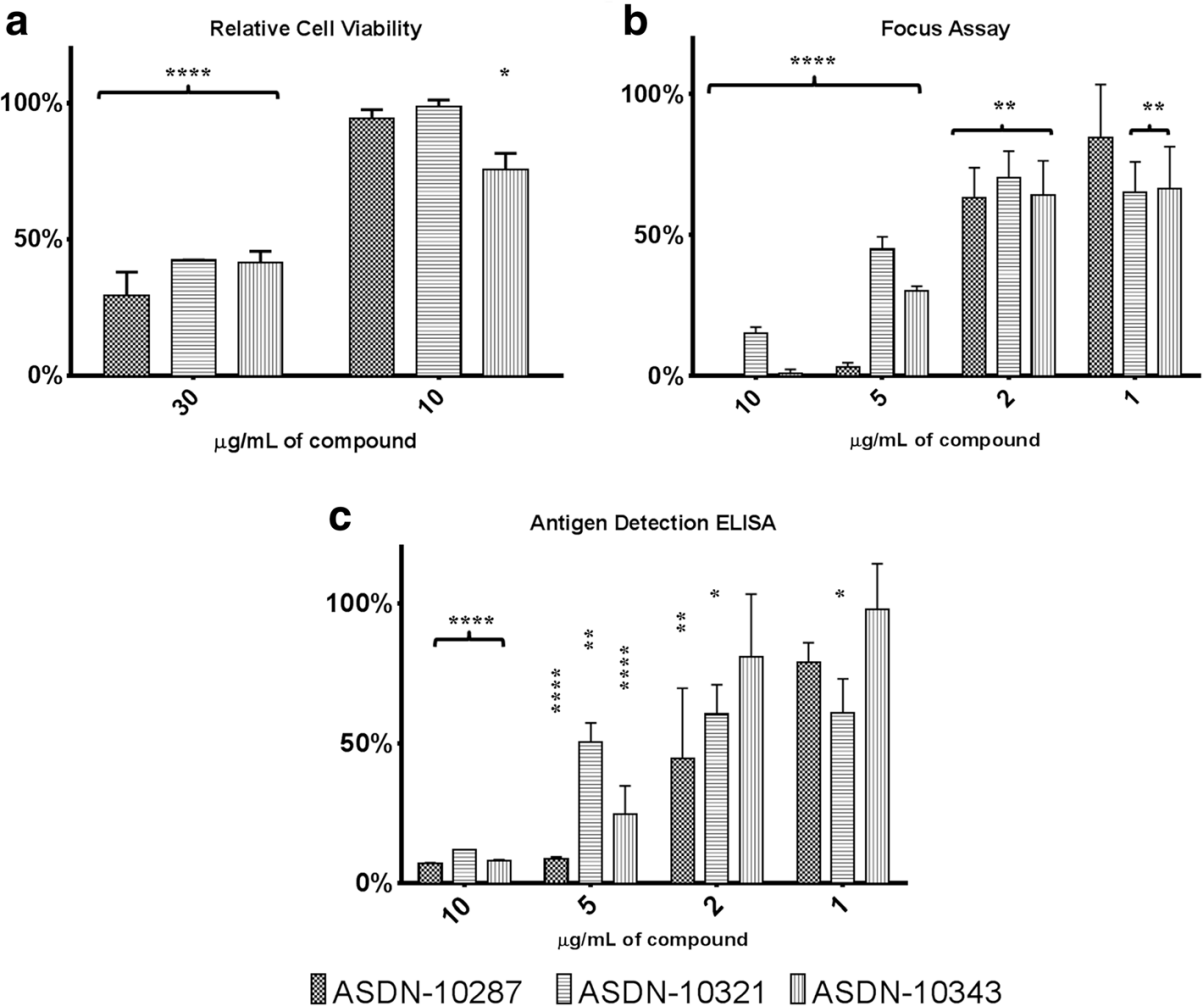
Cell-based assays. **a** Cell viability assay. Effect of compounds on cell viability was observed via the MTT assay. Results are expressed as percentage of live cells relative to vehicle (1 % DMSO) control. **b** Cell-based infectivity assay of compounds. Selected compounds were examined at different concentrations and evaluated for antiviral effect on Vero cells. Activity is reported as percentage relative to infected vehicle-treated only control. **c** Antigen detection ELISA. Effect of the different compounds on the detected viral antigens in culture fluid was examined. Results are expressed as percentage relative to infected vehicle-treated only control. Statistical analysis involved two-way ANOVA with Bonferroni correction. *Error bars* indicate SD

The three compounds were diluted with culture media and added into wells containing Vero cells in confluence prior to detection of formazan formation 72 h post treatment. The results showed cytotoxicity of all compounds at 30 μg/mL. However, there was no significant difference in the cell viability versus 1 % DMSO control with 10 μg/mL except for ASDN-10343 which showed marginal effect (Fig. 5a). Hence, further experiments were done at concentrations starting 10 μg/mL which was considered to be the maximum nontoxic dose (MNTD).

### Cell-based infectivity assays

The effect of the selected compounds on viral infectivity was assessed via focus formation assay. Figure 5b demonstrates the reduction in focus number by the compounds compared to vehicle control (1 % DMSO in culture media). Although the strength of inhibition varied by compound, at least 50 % inhibition at 5 μg/mL minimum concentration was seen among the selected. Significant inhibition was observed at all concentrations tested for ASDN-10321 and ASDN-10343. Qualitative inspection by microscopy of cell growth during the experiments showed congruence with the previous cell viability results (data not shown).

### Antigen detection enzyme-linked immunosorbent assay (ELISA)

The secretion of viral antigens into the culture fluid was also assessed by employing ELISA. Surprisingly, ASDN-10321 also demonstrated significant inhibition at all concentrations tested (Fig. 5c).

## Discussion

The dengue virus protease is a known attractive target for antiviral development. Inhibitors targeting viral proteases have been gaining popularity after recent advances in drug development for hepatitis C and human immunodeficiency viruses, to name a few [33].

Although DENV protease assays have been widely mentioned in literature, it is imperative that the assay conditions be checked and specifically fitted to our objectives. In this study, a biochemical assay for screening of potential inhibitors against dengue protease has been established. The developed assay has identified lead compounds affecting DENV replication which was later confirmed by cell-based infection assays.

We successfully cloned and expressed DENV2 viral protease in *Escherichia coli* and were able to produce the final product with adequate purity for use in an assay. Different isolated peaks were seen during size exclusion chromatography; nonetheless, their SDS-PAGE profiles were similar (Additional file 1: Figure S1). A previous study suggested that this was possibly due to products of premature translation termination and/or existence of aggregated recombinant protease species. Nevertheless, they determined that the different peak fractions did not differ in enzymatic activity [34].

Our study used direct plotting of kinetics data by nonlinear regression wherein estimation is straightforward. It lacks certain disadvantages of linear kinetic plots such as excessive effect of values at the lowest substrate concentrations. The substrate inhibition observed in our study was consistent with the results of Tomlinson et al. using West Nile and dengue viruses [17, 20, 35]. Our study used a fluorogenic substrate based on the results of profiling studies conducted by Li et al. which showed over 100-fold improvement in activity compared to other existing sequences, including those used by Tomlinson [26]. Nevertheless, the inhibition was still observed. This may point out that the length of the substrate and the amino acid sequence interacting with the P3/P4 positions of the enzyme active site may have contributed to this observation. Although the most common sources of possible interference in our observation has been ruled out, other possibilities such as insolubility of end product, intermediary product formation, or pH changes upon substantial substrate cleavage may lead into inefficient release or detection of the fluorescent analyte. Thus, this observation needs to be looked into more closely.

One of the drawbacks of having a standardized single-point screening assay is that there is no account of differences in test compound characteristics. In our study, solubility of some compounds in the aqueous buffer of the protease system was a major difficulty. Visual inspection of the compounds suspended in buffer was necessary to ensure homogeneity during assays. Overtly insoluble compounds were excluded from further analysis. The assay system also suffered problems with autofluorescent compounds which was also a limitation. The drawbacks encountered were not entirely new to drug screening [36–38]. In a study by Simeonov et al., approximately 5 % of the tested library containing 70,000 compounds were more fluorescent than 10 nM of their probe [39].

Recently, recombinant expression of uncoupled NS2B cofactor and NS3 protease has been successful [40, 41]. In vitro fluorescence-based protease assays showed minimal differences between the characteristics of the unlinked and glycine-linked constructs. The most prominent distinction is the apparent lower substrate affinity of the linked construct using longer peptide substrates. They suggested that the linker may have reduced the flexibility of the protease complex therefore restricting access to the active site [40]. This may explain the apparent inhibition observed in our study wherein the excess substrate may have blocked the entry to the site and that the limited flexibility reduced the ability of the protease to form an “induced fit.” Nevertheless, the glycine-linked construct provides an adequate model as target for small, active site inhibitors.

## Conclusions

In this study, we described the successful development of a dengue-2 protease biochemical assay as a useful tool for screening of potential inhibitory compounds. Follow-up experiments of selected compounds confirmed the effects against dengue virus 2 replication in cells. The results will serve as basis for further modifications to the lead compound structure so as to increase its activity against dengue virus and improve its selectivity. Preliminary experiments are currently being undertaken to analogs of the lead compounds as part of a structure–activity relationship study. An opportunity to test the same compounds with other flaviviruses is under consideration.

## Abbreviations

Ac, acetyl; AMC/MCA, 7-amino-4-methylcoumarin; Bz, benzoyl; DENV, dengue virus; DMSO, dimethyl sulfoxide; HCV, hepatitis C virus; HIV, human immunodeficiency virus; HRP, horseradish peroxidase; IC_50_, half maximal inhibitory concentration; IMAC, immobilized metal affinity chromatography; *K*_*m*_, Michaelis constant; MNTD, maximum nontoxic dose; MTT, 3-(4,5-dimethylthiazol-2-yl)-2,5-diphenyltetrazolium bromide; nKRR, norleucine-lysine-arginine-arginine; NS, nonstructural protein/s; OPD, o-phenylenediamine dihydrochloride; PCR, polymerase chain reaction; PVDF, polyvinylidene fluoride; SDS-PAGE, sodium dodecyl sulfate polyacrylamide gel electrophoresis

## Additional file

Additional file 1: Table S1. Primers used in the amplification of the target sequence. Lower case letters denote viral sequence; italicized letters denote sequence comprising the glycine linker region. Letters in bold indicate restriction endonuclease recognition sequences. Figure S1. Fraction collections using size exclusion chromatography. (A) Absorbance detected per each fraction as a function of time. (B) Coomassie SDS-PAGE analysis, (C) anti-histidine Western blot. M = marker, W = 20 mM, 1 = 100 mM, 2 = 200 mM, 3 = 400 mM, 4 = 800 mM Imidazole. Figure S2A. Comparison of different flaviviral protease substrates. Single concentration of substrate (1000 μM) was added into wells containing a fixed amount of DENV2 rNS2B3pro (100 μg/mL) and measured at 30 min. Figure shows relative fluorescence compared to Bz-nKRR-MCA under the same conditions. Error bars show standard deviation (SD). Figure S2B. Protease activity under different glycerol concentrations. Relative protease activity compared to 20 % glycerol standard. Error bars indicate SD. Figure S3A. Inhibition of protease activity by different aprotinin concentrations. Protease activity in the presence of control inhibitor; aprotinin was shown as percentage of enzyme only control. Concentrations ≤10 μM aprotinin significantly differ from the blank (buffer only) at 100 μg/mL recombinant protease concentration. Error bars indicate SD. Figure S3B. A fixed amount of DENV protease was subjected to various concentrations of an inhibitor. Aprotinin, showing trend over time. All sample reaction mixtures contain 100 μg/mL NS2B-NS3pro and 100 μM Bz-nKRR-MCA suspended in 200 mM Tris-HCl with 20 % glycerol, pH 9.5, unless otherwise specified. Error bars indicate standard deviation. (DOCX 1640 kb)
