# Supplementary material for: Development and utility of an in vitro, fluorescence-based assay for the discovery of novel compounds against dengue 2 viral protease
Source: Trop Med Health. 2016 Aug 10;44:22. doi: 10.1186/s41182-016-0025-6 (PMC4979183; doi:10.1186/s41182-016-0025-6)
Supplement: Additional file 1: Table S1. — Primers used in the amplification of the target sequence. Lower case letters denote viral sequence; italicized letters denote sequence comprising the glycine linker region. Letters in bold indicate restriction endonuclease recognition sequences. Figure S1. Fraction collections using size exclusion chromatography. (A) Absorbance detected per each fraction as a function of time. (B) Coomassie SDS-PAGE analysis, (C) anti-histidine Western blot. M = marker, W = 20 mM, 1 = 100 mM, 2 = 200 mM, 3 = 400 mM, 4 = 800 mM Imidazole. Figure S2A. Comparison of different flaviviral protease substrates. Single concentration of substrate (1000 μM) was added into wells containing a fixed amount of DENV2 rNS2B3pro (100 μg/mL) and measured at 30 min. Figure shows relative fluorescence compared to Bz-nKRR-MCA under the same conditions. Error bars show standard deviation (SD). Figure S2B. Protease activity under different glycerol concentrations. Relative protease activity compared to 20 % glycerol standard. Error bars indicate SD. Figure S3A. Inhibition of protease activity by different aprotinin concentrations. Protease activity in the presence of control inhibitor; aprotinin was shown as percentage of enzyme only control. Concentrations ≤10 μM aprotinin significantly differ from the blank (buffer only) at 100 μg/mL recombinant protease concentration. Error bars indicate SD. Figure S3B. A fixed amount of DENV protease was subjected to various concentrations of an inhibitor. Aprotinin, showing trend over time. All sample reaction mixtures contain 100 μg/mL NS2B-NS3pro and 100 μM Bz-nKRR-MCA suspended in 200 mM Tris-HCl with 20 % glycerol, pH 9.5, unless otherwise specified. Error bars indicate standard deviation. (DOCX 1640 kb) [file 41182_2016_25_MOESM1_ESM.docx]

Additional file

| *Primer Name* | *Sequence (5’🡪3’)* |
| --- | --- |
| Nhe1-NS2B-F | TATA**GCTAGC**gccgatttggaactggagag |
| (G4SG4)-NS2B-R | *CCCGCCTCCACCACTACCTCCGCCCCC*cagtgtttgttcttcctcttca |
| (G4SG4)-NS3-F | *GGGGGCGGAGGTAGTGGTGGAGGCGGG*gccggagtattgtgggatgt |
| Xho1-NS3-R | TAAT**CTCGAG**ttactttcggaaaatgtcatcttcg |

**Table S1**. Primers used in the amplification of the target sequence. Lower case letters denotes viral sequence, italicized letters denote sequence comprising the glycine linker region. Letters in bold indicate restriction endonuclease recognition sequences.


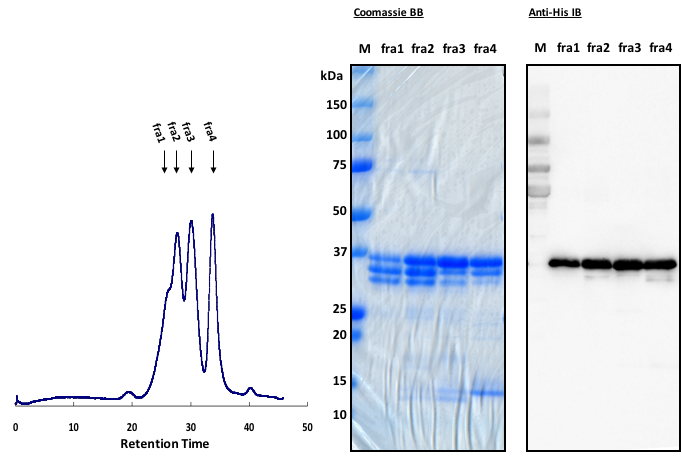


**Figure S1**. Fraction collections using size-exclusion chromatography. (A) Absorbance detected per each fraction as a function of time. (B) Coommassie SDS-PAGE analysis, (C) anti-histidine Western blot. M= Marker, W = 20mM, 1=100mM, 2=200mM, 3=400mM, 4=800mM Imidazole

**Figure S2A**. Comparison of different flaviviral protease substrates. Single concentration of substrate (1000µM) were added into wells containing fixed amount of DENV2 *rNS2B3pro* (100µg/mL) and measured at 30 minutes. Figure shows relative fluorescence compared to Bz-nKRR-MCA under the same conditions. Error bars show standard deviation (SD)

**Figure S2B**. Protease activity under different glycerol concentrations. Relative protease activity compared to 20% glycerol standard. Error bars indicate SD.

**Figure S3A**. Inhibition of protease activity by different aprotinin concentrations. Protease activity in the presence of control inhibitor, aprotinin were shown as percentage of enzyme only control. Concentrations ≤10µM aprotinin significantly differ from the blank (buffer only) at 100µg/mL recombinant protease concentration. Error bars indicate SD.

**Figure S3B**. A fixed amount of DENV protease was subjected to various concentrations of an inhibitor, Aprotinin, showing trend over time.

All sample reaction mixtures contain 100μg/mL NS2B-NS3pro and 100μM Bz-nKRR-MCA suspended in 200mM Tris-HCL with 20% Glycerol pH 9.5 unless otherwise specified. Error bars indicate Standard Deviation.
